# National Cancer Institute Imaging Data Commons: Toward Transparency, Reproducibility, and Scalability in Imaging Artificial Intelligence

**DOI:** 10.1148/rg.230180

**Published:** 2023-11-24

**Authors:** Andrey Fedorov, William J. R. Longabaugh, David Pot, David A. Clunie, Steven D. Pieper, David L. Gibbs, Christopher Bridge, Markus D. Herrmann, André Homeyer, Rob Lewis, Hugo J. W. L. Aerts, Deepa Krishnaswamy, Vamsi Krishna Thiriveedhi, Cosmin Ciausu, Daniela P. Schacherer, Dennis Bontempi, Todd Pihl, Ulrike Wagner, Keyvan Farahani, Erika Kim, Ron Kikinis

**Affiliations:** From the Department of Radiology, Brigham and Women’s Hospital and Harvard Medical School, 399 Revolution Dr, Somerville, MA 02145 (A.F., D.K., V.K.T., C.C., R.K.); Institute for Systems Biology, Seattle, Wash (W.J.R.L., D.L.G.); General Dynamics Information Technology, Rockville, Md (D.P.); PixelMed Publishing, Bangor, Pa (D.A.C.); Isomics, Cambridge, Mass (S.D.P.); Departments of Radiology (C.B.) and Pathology (M.D.H.), Massachusetts General Hospital and Harvard Medical School, Boston, Mass; Fraunhofer MEVIS, Bremen, Germany (A.H., D.P.S.); Radical Imaging, Boston, Mass (R.L.); Artificial Intelligence in Medicine Program, Mass General Brigham, Harvard Medical School, Boston, Mass (H.J.W.L.A., D.B.); Radiology and Nuclear Medicine, CARIM & GROW, Maastricht University, Maastricht, the Netherlands (H.J.W.L.A., D.B.); Frederick National Laboratory for Cancer Research, Rockville, Md (T.P., U.W.); and National Cancer Institute, Bethesda, Md (K.F., E.K.).

## Abstract

The remarkable advances of artificial intelligence (AI) technology are revolutionizing established approaches to the acquisition, interpretation, and analysis of biomedical imaging data. Development, validation, and continuous refinement of AI tools requires easy access to large high-quality annotated datasets, which are both representative and diverse. The National Cancer Institute (NCI) Imaging Data Commons (IDC) hosts large and diverse publicly available cancer image data collections. By harmonizing all data based on industry standards and colocalizing it with analysis and exploration resources, the IDC aims to facilitate the development, validation, and clinical translation of AI tools and address the well-documented challenges of establishing reproducible and transparent AI processing pipelines. Balanced use of established commercial products with open-source solutions, interconnected by standard interfaces, provides value and performance, while preserving sufficient agility to address the evolving needs of the research community. Emphasis on the development of tools, use cases to demonstrate the utility of uniform data representation, and cloud-based analysis aim to ease adoption and help define best practices. Integration with other data in the broader NCI Cancer Research Data Commons infrastructure opens opportunities for multiomics studies incorporating imaging data to further empower the research community to accelerate breakthroughs in cancer detection, diagnosis, and treatment.

Published under a CC BY 4.0 license.

## Graphical abstract

**Figure:**
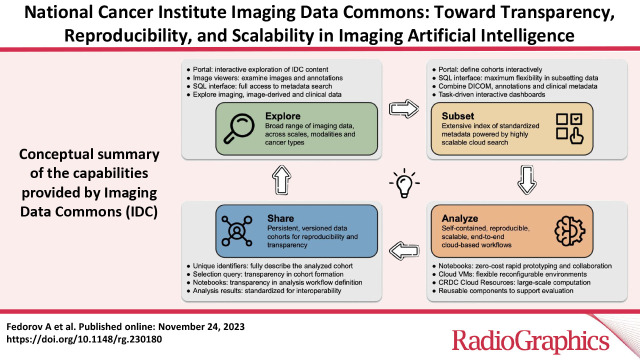

## Introduction

The remarkable advances of artificial intelligence (AI) technology are revolutionizing established approaches to the acquisition, interpretation, and analysis of biomedical imaging data. Many promising AI-based tools have been introduced both in the clinic and in the laboratory (1). Development, continuous refinement, and validation of such tools require easy access to large high-quality annotated datasets that are both representative and diverse (2). Establishing such datasets in the field of medical imaging comes with numerous complexities (3). Acquisition of medical images requires highly specialized and complex equipment and personnel. Specialized expertise and significant effort are required to correctly de-identify and curate such data (4). Storage and retrieval of large imaging datasets can present additional challenges, as does orchestrating computation on this scale.

The National Cancer Institute (NCI), part of the U.S. National Institutes of Health, has invested significant resources into the collection of large amounts of health-related data, including imaging (5–8). Efforts were undertaken to support de-identification, curation, and access to the imaging data with the introduction of The Cancer Imaging Archive (TCIA) about 10 years ago (9). With the nascent effort to establish the national cancer data ecosystem, as one of the priorities for the Cancer Moonshot (10), the emphasis is shifting beyond supporting data archival and access and toward enabling the collaborative use and analysis of these datasets within data commons (11). A core component of the ecosystem is the NCI Cancer Research Data Commons (CRDC)—a cloud-based data science infrastructure that provides secure access to a large, comprehensive, and expanding collection of cancer research data, along with the analytical and visualization tools for data analysis across domains.

NCI Imaging Data Commons (IDC) (12) (*https://imaging.datacommons.cancer.gov/*) is a component of the CRDC infrastructure that hosts publicly available cancer imaging data colocated with analysis and exploration resources. Since the initial release in 2020, the platform has been evolving, expanding both the data offering and the capabilities supporting data use. Today, IDC is an established imaging data science platform, providing de-identified Digital Imaging and Communications in Medicine (DICOM) imaging data and metadata, analysis results collections, capabilities for viewing, cohort selection, and downstream AI and machine learning (ML) development and analysis using customized or cloud-native tools (13). IDC collections are publicly available, versioned and harmonized into DICOM representation, to meet Findable, Accessible, Interoperable, Reusable (FAIR) principles (14).

IDC is uniquely positioned to help improve transparency, reproducibility, and scalability of the emerging AI analysis tools in biomedical imaging. Lack of transparency (the availability of details accompanying the analysis to enable scientific understanding of how the analysis was performed [15]) and limited reproducibility (the ability to replicate the analysis given the same input data [16]) of AI imaging analysis workflows is broadly recognized as a major obstacle to clinical translation (15). Scalability characterizes the system as being capable of efficiently supporting increasing load sizes. Given both the growing computational complexity of the modern AI algorithms and the sizes of imaging datasets, scalability becomes a critical attribute to enable evaluation and application of imaging AI advances in practice.

In this article, we summarize the key implementation principles of IDC, highlight its current features and capabilities, and discuss major developments and updates since its initial release. As a data commons, IDC enables its users to explore and analyze data and share the generated analysis results. To illustrate this, we highlight some of the recent applications and projects utilizing IDC, with the emphasis on how IDC is positioned to enable its users to improve transparency, reproducibility, and scalability of their analyses.

## Overview of NCI IDC

As of the writing this article, the IDC contains over 67 TB (including prior versions) of imaging data spanning a range of image acquisition techniques (eg, radiology imaging modalities, digital pathology, and multiplexed fluorescence imaging) and devices, cancer and tissue types, and organ systems. There are several aspects of the IDC that differentiate it from other repositories.

### DICOM for Data Harmonization

The core underpinning of the FAIR Data Principles is in “enhancing the ability of machines to automatically find and use the data, in addition to supporting its reuse by individuals” (14). To achieve this vision, it is mandatory that metadata follows consistent conventions, which necessitates the use of a standard. All of the images and image-derived data (ie, annotations, segmentations of the regions of interest, image-derived features, analysis results) hosted by IDC are natively encoded as, or harmonized into, DICOM representations (13). In situations where data are supplied in a research format or vendor-specific representation, conversion of the data into DICOM is done by the IDC team. While DICOM was originally developed to support clinical workflows focused on radiology, it has demonstrated utility to support other imaging types and applications (17,18) by enabling interoperability and providing a consistent data model and metadata conventions. DICOM representation is rich with metadata (both structured and unstructured) that can enable searching and processing of the images. DICOM enables interoperability, which means that IDC can use off-the-shelf tools (both commercial and open source) implementing the standard, reducing development and maintenance costs, and supporting reuse of the analysis workflow components. There is no alternative standard for harmonizing representation of pixel data and metadata that can address the breadth of use cases in medical imaging.

### Cloud-based Hosting of Data

IDC data are hosted using public cloud services to streamline exploration, search, and analysis of the data (as we discuss in the example use cases later in this article), while leveraging flexibility of the cloud to enable security and scalability. IDC relies on the services provided by both Google Cloud Platform (GCP; Google) and Amazon Web Services (AWS; Amazon). While hosting IDC data in the cloud makes it easier to use cloud-based tools, users can also use their own computational resources for analyzing IDC data.

### Public Availability of All Data

All of the data hosted by IDC are available publicly and can be downloaded for either cloud-based or on-premises analysis. Most of the collections in IDC are governed by nonrestrictive licenses allowing use of the images in research and in development of medical products. A small number of collections in IDC limit use to noncommercial activities.

### Data Versioning

As the content of individual collections evolves, IDC provides persistent access to the prior versions of each file. Data can be removed from IDC under rare and exceptional circumstances, such as the retraction of the dataset, for example, if protected health information (PHI) is discovered in the data.

### Balance of Open-Source and Commercial Components

IDC is implemented using a combination of open-source and commercial tools. Commercial offerings from the leading cloud providers are used to enable scalability, competitive pricing for the access to cloud-based resources, and manageable operational costs. Open-source components enable support of the evolving needs of the cancer imaging research community.

### Data Acceptance Criteria

To deposit a dataset to IDC, the submitters must establish its quality and scientific value (eg, by demonstrating the collection is supported by a funded initiative, or it is accompanied by a peer-reviewed article). Data must be de-identified before depositing to IDC. Unless the data are de-identified by an entity that is approved by NCI Security, the submitter must complete risk mitigation documentation describing de-identification approaches and procedures to follow in case PHI is discovered in the data. Contributors must be comfortable with the public (as opposed to restricted) release of the dataset. Finally, the dataset must be released under a permissive license. Most of the data in IDC are covered by the Creative Commons By Attribution license, which permits commercial use of the data. Resources of the IDC team to harmonize incoming data are always going to be limited. Assuming these criteria are satisfied, prioritization of a specific dataset for ingestion is determined by the IDC stakeholders. We expect this procedure to be refined to better address the evolving needs of the community in implementing the recently introduced National Institutes of Health Data Management and Sharing Policy.

## Content, Capabilities, and Intended Users

### Content

Initially IDC focused on ingesting public DICOM radiology collections already de-identified and curated by TCIA (9). TCIA continues to be an active partner of IDC: data contributors are encouraged to submit images to TCIA with a well-defined pathway to making the data available in IDC. Beyond these initial radiology collections from TCIA, IDC proceeded to ingest digital pathology components of the data collected by The Cancer Genome Atlas (TCGA), The Clinical Proteomic Tumor Analysis Consortium (CPTAC) (6), and the National Lung Screening Trial (NLST) (8), some of which were also hosted in proprietary formats by TCIA. Harmonization of the initial release of the imaging data collected by the Human Tumor Atlas Network (HTAN) (7), including multichannel fluorescence images, was the next important milestone. Importantly, the tools and procedures used by the IDC team to perform conversion into DICOM representation are documented and publicly available, paving the path for broader adoption of DICOM.

In most applications, meaningful interpretation and analysis of images can be challenging without the data describing the clinical characteristics of the patient. Such information is often stored in nonstandardized attachments that accompany the images. To better meet the FAIR principles for the accompanying clinical data, we implemented curation procedures that parse such attachments and ingest clinical metadata into IDC, along with the dictionaries describing their content (when available). All of the clinical data in IDC is searchable using the Standard Query Language (SQL) interface and is linked with the imaging data via the patient or case identifiers.

**Figure 1.**
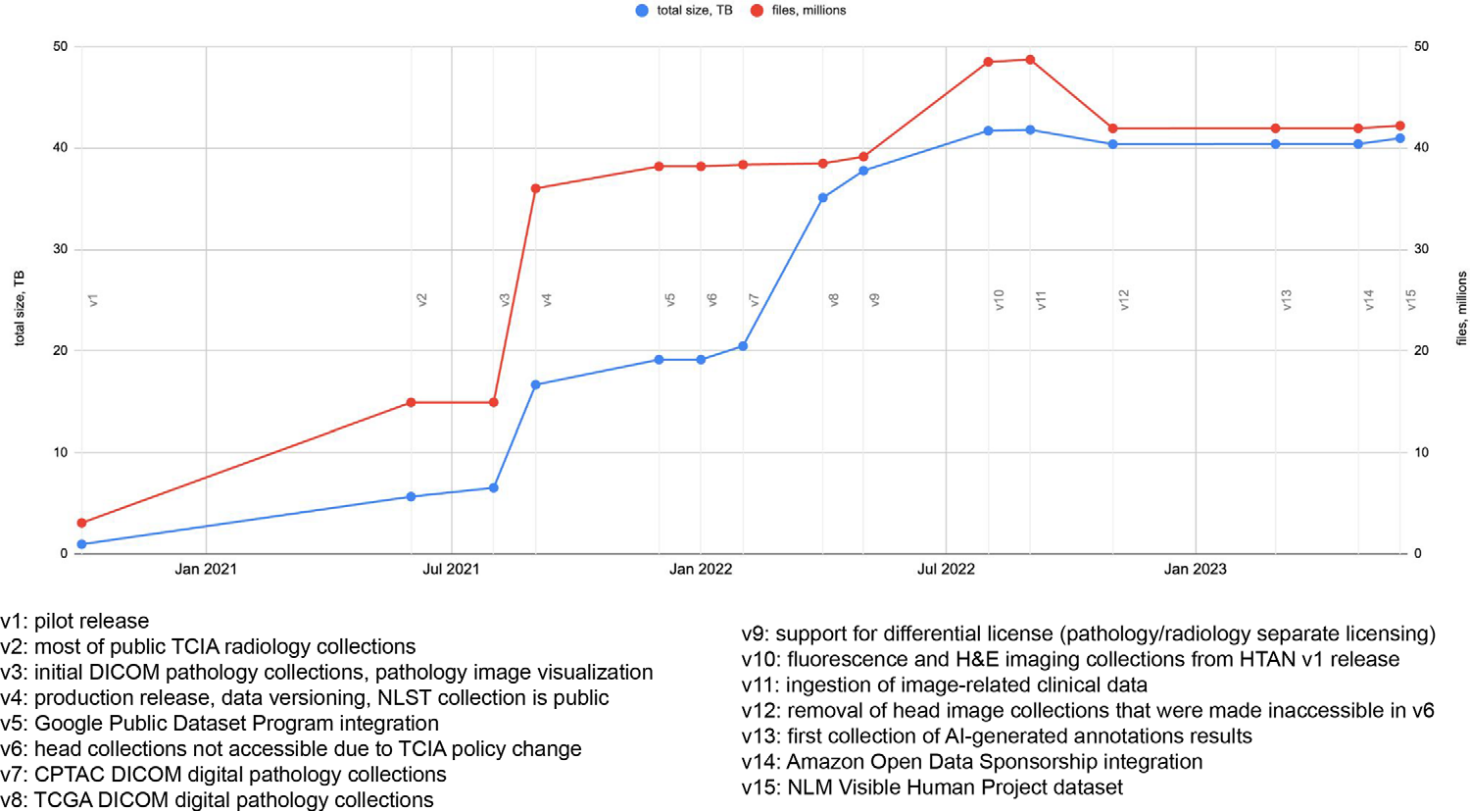
Annotated timeline of IDC data releases and major development milestones. An up-to-date version of the timeline is available in the IDC data release notes documentation page (19). CPTAC = The Clinical Proteomic Tumor Analysis Consortium, H&E = hematoxylin and eosin, HTAN = Human Tumor Atlas Network, NLM = National Library of Medicine, NLST = National Lung Screening Trial, TB = terabyte, TCGA = The Cancer Genome Atlas.

**Figure 2.**
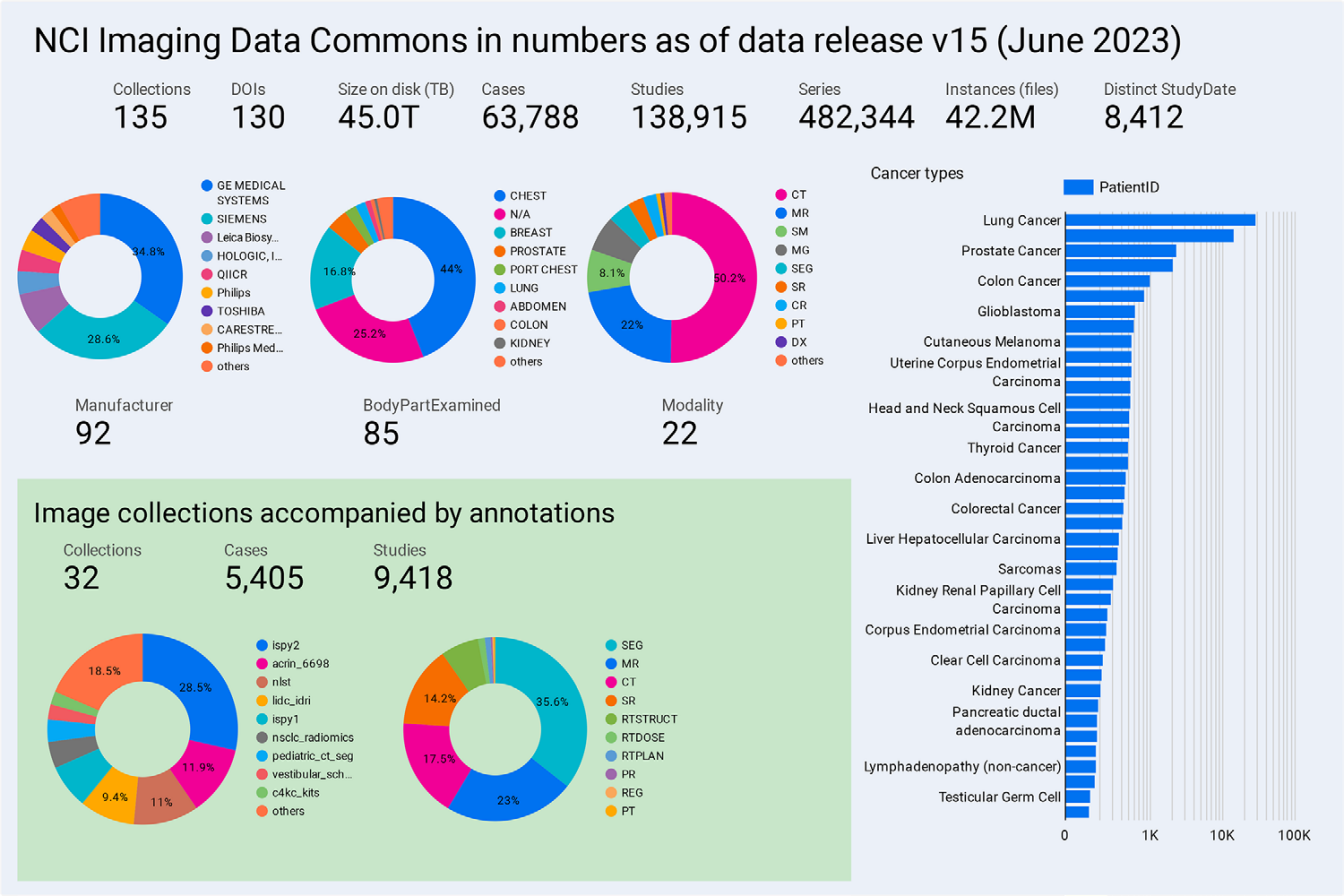
Chart shows a summary of the data available in IDC as of data release version 15 (June 2023). Note that the size on disk reported is in terabytes (TB) (10^12^ bytes). An interactive version of this summary dashboard is publicly available (21).

**Figure 3.**
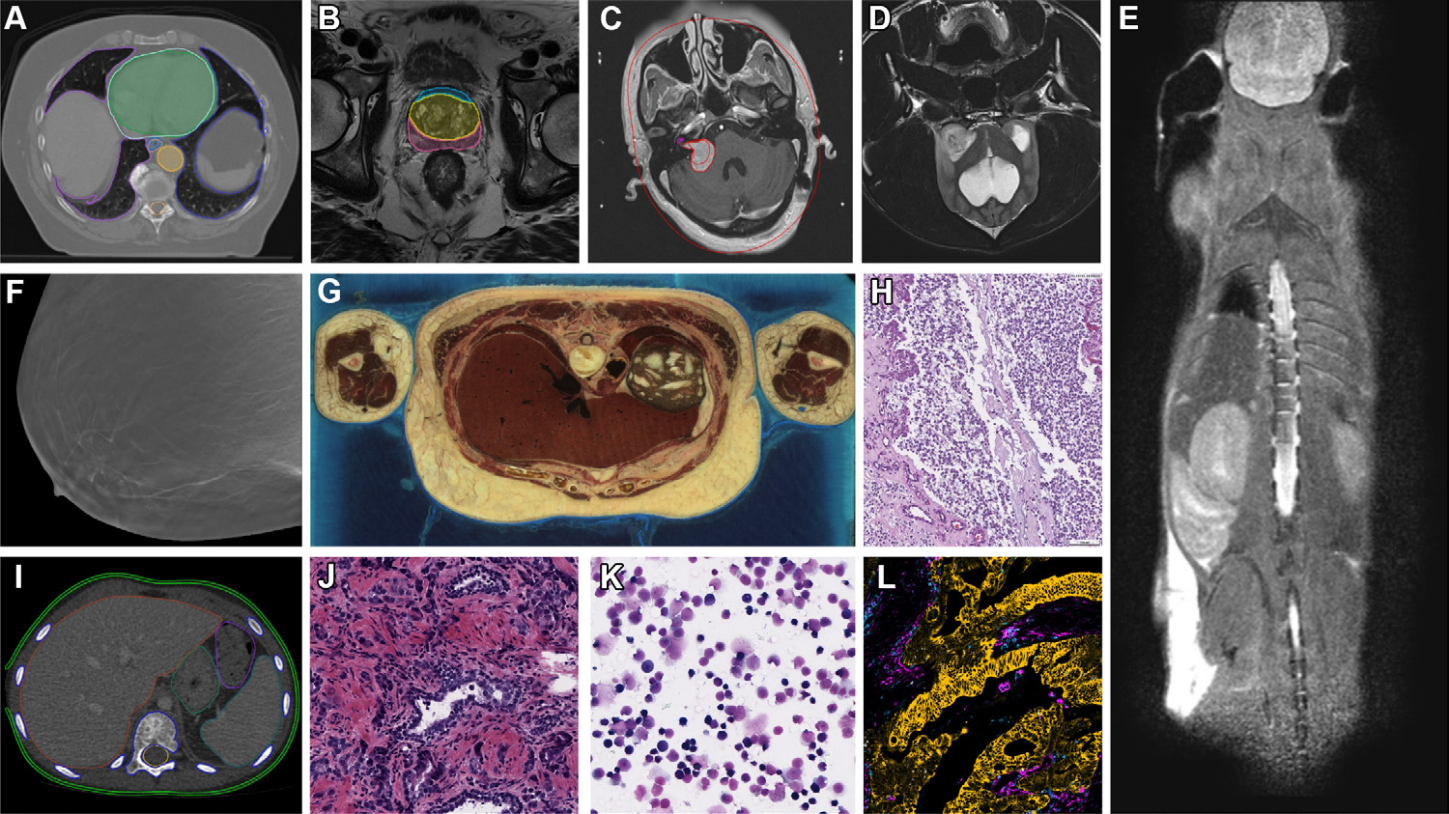
Representative images and image annotations available in IDC. **(A)** NSCLC-Radiomics (22) CT image shows lung cancer with manually annotated regions of interest and nnU-Net-BPR-Annotations (23) (AI-annotated regions of interest). *NSCLC* = non–small cell lung cancer. **(B)** PROSTATEx (24) MR image shows PROSTATEx-Seg-Zones (25), expert-annotated prostate anatomy zones. *Seg* = segmentation. **(C)** Vestibular-Schwannoma-SEG (26) MR image shows schwannoma with manually annotated regions of interest. **(D)** ICDC-Glioma (27) MR image shows canine glioma. *ICDC* = Integrated Canine Data Commons. **(E)** PDMR-997537-175-T MR image shows a mouse adenocarcinoma colon xenograft. *PDMR* = Patient-Derived Models Repository. **(F)** Breast-Cancer-Screening-DBT (28) tomosynthesis image. *DBT* = digital breast tomosynthesis. **(G)** NLM-Visible-Human-Project (20) cryomacrotome anatomic image. **(H)** ICDC-Glioma (28) canine hematoxylin and eosin (H-E) stain digital pathology photomicrograph. **(I)** Pediatric-CT-SEG (29) pediatric CT image with expert-annotated organ contours. **(J)** TCGA-PRAD (5) H-E stain digital pathology photomicrograph shows prostate cancer. *TCGA-PRAD* = The Cancer Genome Atlas Prostate Adenocarcinoma. **(K)** CPTAC-AML (6) H-E stain digital pathology photomicrograph shows acute myeloid leukemia. *CPTAC-AML* = Clinical Proteomic Tumor Analysis Consortium Acute Myeloid Leukemia. **(L)** HTAN-HMS (7) multichannel fluorescence image with pan-cytokeratin, CD45, vimentin, and Ki67 channels selected. *HTAN* = Human Tumor Atlas Network.

As of data release version 15 (June 2023; the annotated timeline of releases is shown in Fig 1), IDC contains most of the public radiology collections from TCIA and a range of collections that are unique to IDC. Such IDC-specific content includes DICOM-converted digital pathology collections, originally distributed in a vendor-specific format by the TCGA, CPTAC, NLST, and HTAN initiatives, and the National Library of Medicine (NLM) Visible Human Project (20) dataset, which until recently was only available in a proprietary vendor format from NLM. Current content of IDC is summarized in Figure 2, with a sample of highlight images shown in Figure 3.

### Capabilities

**Figure 4.**
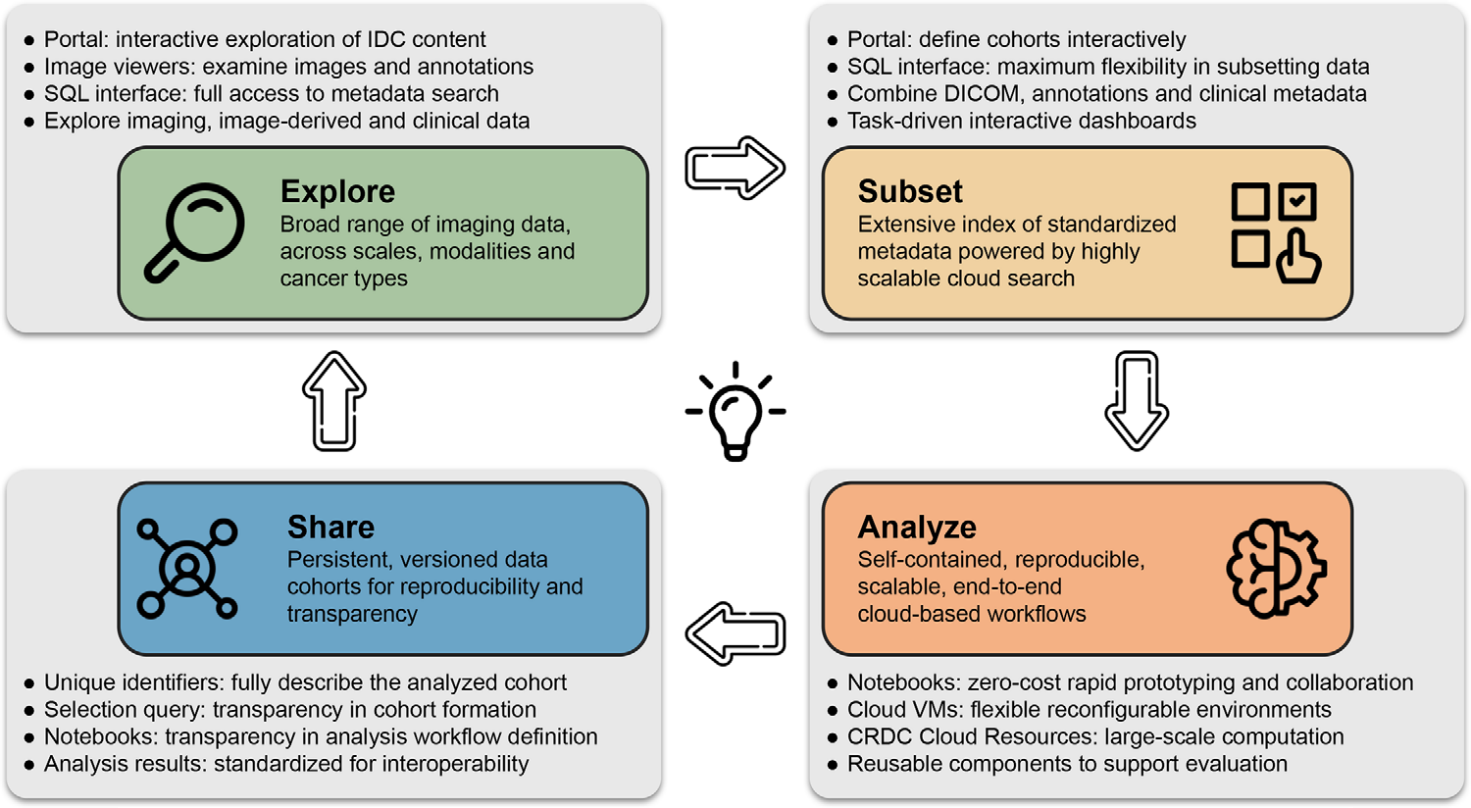
Conceptual summary of the capabilities provided by IDC and flowchart of the interactions of the target user with the platform. Each of the gray panels highlights the specific components available within IDC to support the corresponding capabilities. *VM* = virtual machine.

IDC is an actively maintained data commons that is continuously growing to include new cancer imaging data collections but also is a resource to support interaction with and use of the data. We envision the following broad categories of activities and interactions with the data that IDC can enable, as summarized in Figure 4.

**Explore.—**Conversion of the data into DICOM representation enables basic uniformity of the data model and metadata. This in turn makes it possible to use consistent selection criteria while interacting with the data. Datasets sharing common metadata expressing relationships can be cross-linked into graphs representing, for example, which input images and human annotations were used as input to ML algorithms. The IDC web portal application provides the entry-level interface to enable exploration of IDC data. Users seeking more detailed data can use the SQL interface, which provides complete access to both the collection-level and DICOM metadata describing the files hosted by IDC and to the clinical data accompanying those collections. Images and accompanying annotations can be visualized using hosted instances of Open Health Imaging Foundation (OHIF; *https://ohif.org/*) (radiology images) and Slim (*https://github.com/ImagingDataCommons/slim*) (digital pathology and fluorescence images) viewers (30, 31).

**Subset.—**IDC enables unambiguous referencing of individual items and cohorts using unique identifiers. IDC data are versioned, and such references will remain valid and will point to the same files, unaffected by the updates to the IDC content. Using either the IDC portal or SQL queries, comprehensive metadata-based selection criteria filters can be used to define cohorts or subsets of data for a specific analysis task. Application of a query filter to a specific version of IDC data can be used to precisely and reproducibly define the list of files corresponding to the selection.

**Analyze.—**Standard representation of the data and its colocation with the scalable cloud-based computational resources have the potential to lower the barriers for the analysis of IDC data. Data loading and preprocessing workflows can be standardized and leverage existing libraries implementing DICOM support and can be applied to any standard dataset. Today, all major cloud-computing vendors provide integration of Jupyter notebooks (32), under different products, hosted on seamlessly provisioned cloud virtual machines.

Combined with efficient access to the cloud-based image data, such notebook environments can be used to quickly apply existing tools and pipelines to selected IDC datasets and define the workflow for a larger cohort analysis. Highly scalable computational resources for the analysis of those cohorts can be provisioned directly from the cloud providers or by using platforms, such as Terra (*https://terra.bio/*) and Seven Bridges Cancer Genomics Cloud (*https://www.cancergenomicscloud.org/*), that implement additional layers of abstraction (33, 34).

**Share.—**The definition of the cohort that was selected from IDC and used in an analysis can be unambiguously recorded and shared to help achieve transparency. Analysis workflows can be shared in a form that will enable recipients to re-execute the workflow in a reproducible manner, with the cloud environments simplifying the provisioning of the precise environment (both in terms of the virtualized hardware and software required). Visualization of the data used in the analysis and exploration of the accompanying metadata can be accomplished with minimal effort by the recipient using the infrastructure maintained by IDC through simply sharing a web link. The aforementioned resources aim to complement and improve the quality, rigor, accessibility, and reproducibility of the traditional academic publications. Importantly, outputs produced in the process of analyzing IDC data can be harmonized into appropriate DICOM objects and contributed back to IDC, enriching its content and allowing more rapid development of the analysis tools.

### Intended Users

We envision the primary group of IDC users to be biomedical computational scientists interested in cancer research who have a technical background in computer science, informatics, or related fields. The content and capabilities of IDC expect the user to have at least some understanding of biomedical imaging. Increasing availability of image-derived features in IDC (eg, shape and intensity texture features characterizing tissue patterns or morphology) aims to make it easier to perform hypothesis exploration and multiomics analysis of IDC data by users with less imaging expertise.

There are many uses of the data IDC contains and IDC infrastructure that could be of interest for the broader audience. Early-stage scientists and students, especially those who are not part of the established groups positioned to have access to large institutional repositories, should be able to identify data that may be relevant to their areas of interest and evaluate existing state-of-the-art analysis tools, prototype, collaborate, and share intermediate findings. Articles presenting novel analysis tools or imaging-based findings can be accompanied by computational notebooks or demonstrations of the developed tools, if not complete containerized analysis workflows. This can be of benefit to academic researchers as well as publishers seeking to make publications more accessible and attractive.

Developers of commercial solutions can evaluate proprietary tools and benchmark them against state-of-the-art open-source solutions or use the data in demonstrations and pilot projects. Funders can consider recommending the use of IDC as a persistent repository holding images and analysis results for broader dissemination. IDC can also simplify the task of evaluating continuously evolving AI tools for practicing radiologists with interest in imaging research. Benchmarking of such tools made readily available by using the cloud resources against public datasets can streamline selection of robust tools before their further evaluation on the internal datasets. Availability of annotations and ongoing work to enrich existing collections with AI-derived annotations, measurements, and features can be of further interest to clinical users, including radiologists, pathologists, and other specialists.

## Use Cases

In this section, we illustrate the capabilities of IDC discussed previously with their application to address specific needs in the context of biomedical imaging research, while enabling transparency, reproducibility, and scalability of the analyses. While those activities are somewhat interrelated, we discuss them to draw the attention of different communities of cancer researchers and technology developers.

### Best Practices for Data Provenance in Research Reports

Insufficient or unknown provenance of the datasets used while developing ML imaging tools plagues many, if not most, research studies (35), in turn jeopardizing transparency of those studies and reducing their reproducibility. Even with the best intent, data provenance reporting is fraught with pitfalls in the absence of an easily accessible, machine-readable, and detailed description of the dataset.

**Figure 5.**
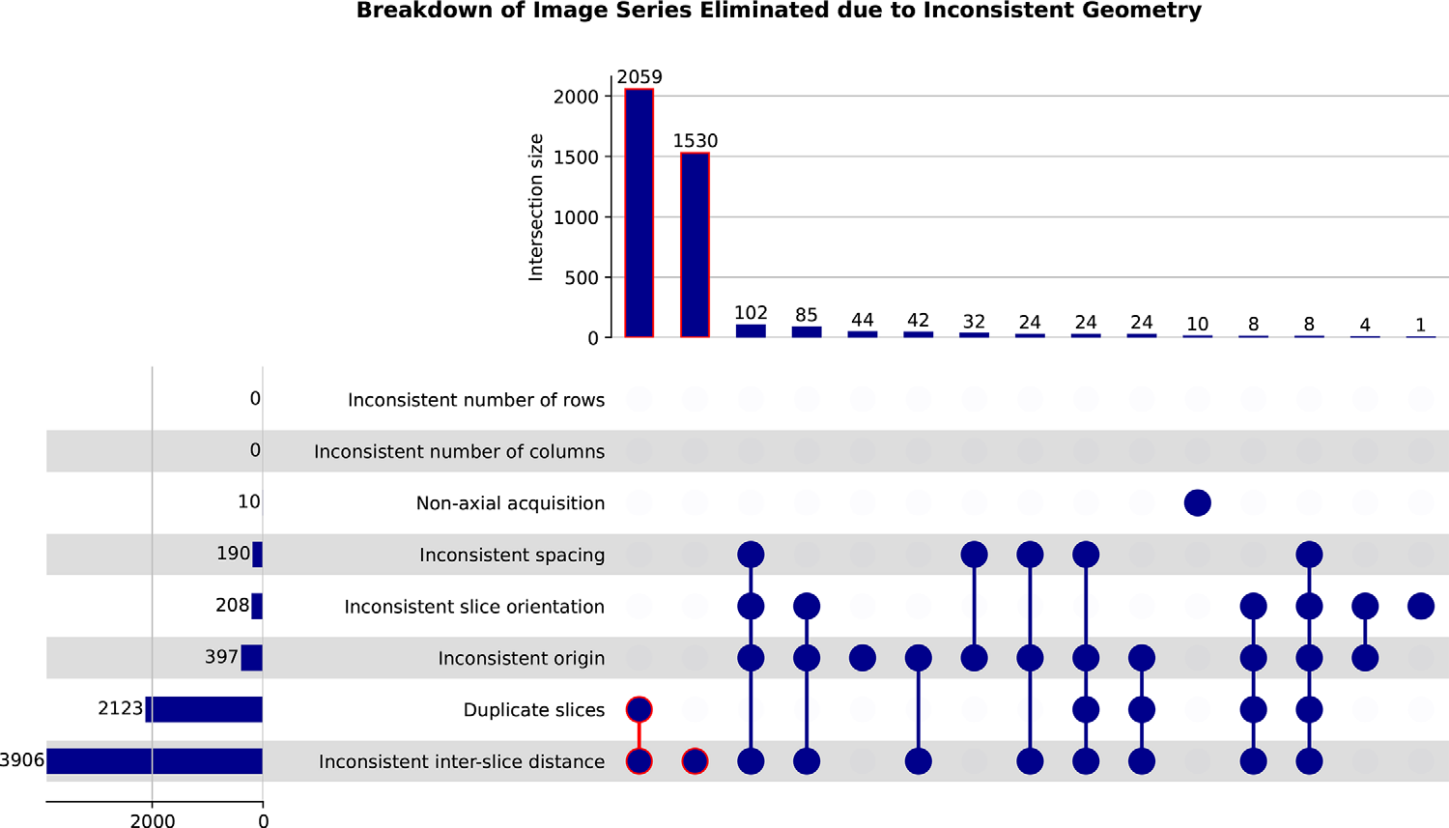
UpSet plots show the distributions of DICOM series from the National Lung Screening Trial (NLST) collection that were identified to have inconsistent geometry. Definition of the rules to identify these series was done by using an SQL statement against the DICOM metadata available in IDC. Items outlined in red correspond to the DICOM series groups constituting the largest portion of those that have inconsistent geometry.

IDC offers a practical means to address the FAIR principles while describing a dataset used in training or benchmarking an analysis tool. Every single file in IDC is assigned a unique and persistent identifier, with the revisions of the file assigned new identifiers and tracked by IDC versioning. Accompanying metadata is available in a standard representation and is searchable by using a standardized communication protocol, allowing anyone to assess its heterogeneity and the presence of biases. The data and metadata are maintained as close to the source representation as possible. Availability of such metadata can reduce the effort and increase the transparency of data curation, allowing, for example, identification of images that are not suitable for use with a specific AI model without downloading the data (Fig 5). When converting into a DICOM representation, accompanying metadata is harmonized and, where possible, enriched by including additional attributes describing acquisition, as an example. A dataset defined as a list of IDC unique identifiers, accompanied by the selection query used to produce it, is a persistent solution for dataset reporting.

### Reproducibility of Scientific Studies

Reproducibility, replicability, and repeatability (36–38) prove to be challenging when developing AI tools for medical imaging applications (15). In our experience, IDC and cloud infrastructure can simplify the process of achieving each (39). The hardware configuration and software stack can be described in a manner that allows anyone to instantiate a virtual machine replicating the original deployment environment. When development or evaluation of an analysis method uses public data from IDC, cloud resources combined with versioned code repositories and the aforementioned dataset descriptions can effectively complement the traditional article describing the study.

**Figure 6.**
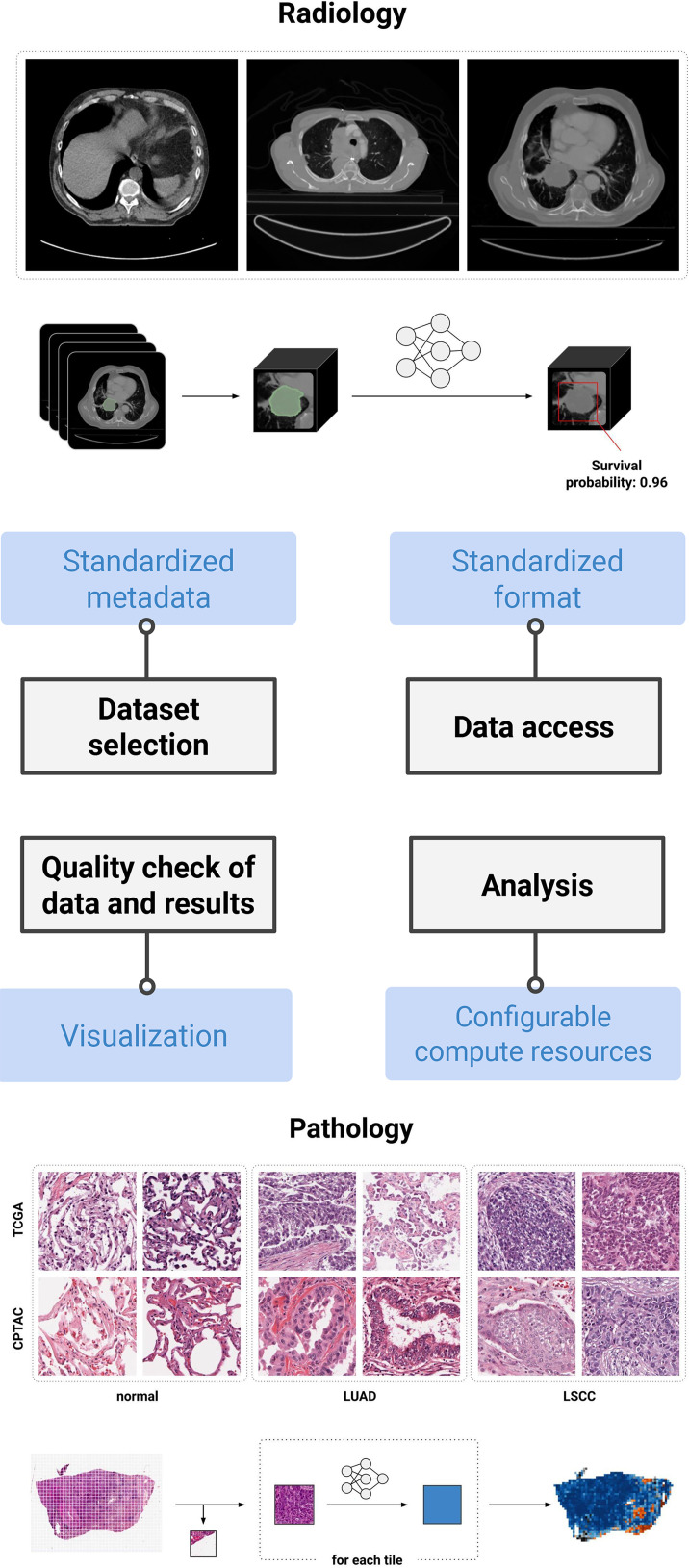
Highlights of IDC features and their contribution to the development of reproducible pipelines. To date, two reproducible analysis studies have been conducted by the IDC team: automated prognosis of lung cancer mortality based on standard-of-care CT (40) and automated classification of lung cancer histopathologic images (39). Both are accompanied by notebooks allowing for reproduction if the study is using the cloud-provisioned compute resources and data available at IDC. *LSCC* = lung squamous cell carcinoma, *LUAD* = lung adenocarcinoma.

In situations where analysis involves nonpublic datasets, it may be possible to find a sufficiently similar dataset in IDC that can be used to provide reproducible demonstrations. An example might be to demonstrate the utility of segmentation tools developed on institutional data when applied to IDC public data. Recent studies investigated the utility of IDC in improving reproducibility of specific AI studies in radiology (40) and digital pathology (39) (Fig 6).

### Accessibility and Transparency of Image Analysis Tools

We define *accessibility* of an image analysis tool as the ability of a user, with the minimal effort, to apply this tool to a demonstration dataset where the tool is expected to perform well and further to any dataset that meets the input requirements. Accessibility of a tool can be dramatically improved if the article describing the tool and its documentation are accompanied by working examples that incorporate all of the steps needed to deploy and configure the tool, preprocess the data, perform the analysis on a representative dataset, visualize the results, etc. Such minimal working examples are similar to the concept of a *software vignette* first introduced in the Bioconductor project (41).

IDC can serve as the source of readily accessible and searchable public image data that can support such notebooks in medical imaging research. As an example, IDC tutorial materials include a notebook that demonstrates the end-to-end process of performing inference on a chest CT examination using the nnU-Net Task055 SegTHOR (Segmentation of THoracic Organs at Risk) segmentation model (42). Such a minimal example is useful well beyond a mere demonstration of functionality. By making minor adjustments to the selection query, users can experiment with applying the tool to datasets from a variety of collections and manufacturers, gathering hands-on experience with generalizability of the tool. The example operates on the data in the DICOM format, as collected by the imaging equipment used in the clinic, which makes it easier to experiment with the same tool on nonpublic institutional data. This creates opportunities to dramatically improve the scholarly publications by accompanying them with working examples that are relatively easy to create and even easier for readers of the article to reuse.

### Enrichment of Public Imaging Datasets

Analysis of medical imaging data and, in particular, integration of imaging data with other sources of biologic data often involves intermediate processing operations, such as segmenting a region of interest or biomarker quantification. Image annotations, which can be pixel-level labeling of the region of interest or image-level assignment of a label, can also be instrumental in making imaging data more searchable and reusable. In such usage scenarios, it is of significant value to have datasets that are accompanied by the annotations and other image-derived items produced by experts and established automated analysis tools. The application of existing tools on public data could be considered by some as straightforward in principle. In practice, access to specialized hardware and domain expertise is required to deploy and configure the tool, address variations in the input data, perform analysis in a time- and cost-efficient manner, conduct quality checks, and document the process and the result.

**Figure 7.**
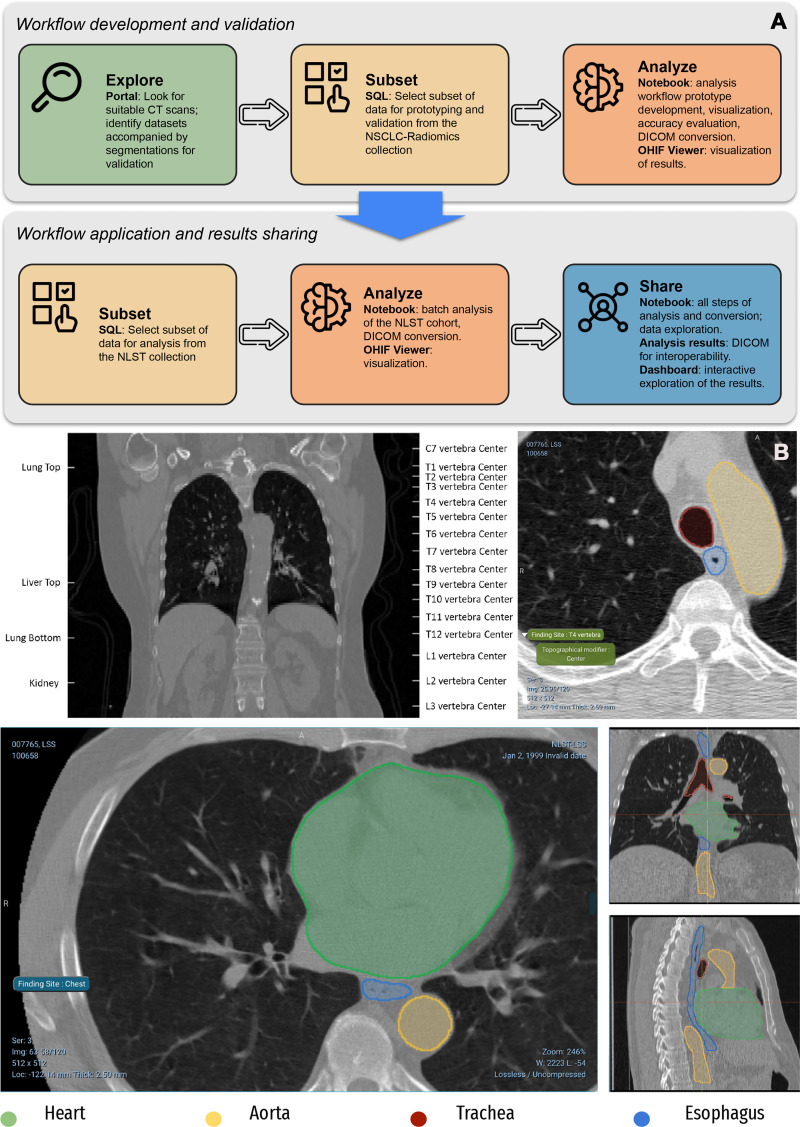
AI-generated annotations of CT images using BodyPartRegression (43) and the nnU-Net Task055 SegTHOR segmentation model (42), with further details on the application of these algorithms to the NSCLC-Radiomics and NLST collections of IDC discussed by Krishnaswamy et al (23). **(A)** Top panel of the flowchart shows the process of development and interaction with the individual components of IDC. The bottom panel of the flowchart shows products generated in the process of analysis. **(B)** Top CT images containing anatomic landmarks corresponding to the centers of vertebra and several other structures (top left) are identified and labeled automatically (top right). Bottom CT images show volumetric segmentations of the pixels corresponding to the heart, esophagus, aorta, and trachea.

As described in a recent study (23), IDC can support development of the analysis pipelines aimed at enriching existing datasets (Fig 7). Resulting pipelines can be developed following the examples mentioned in the previous sections to facilitate their transparency and reproducibility. Availability of the resulting annotations along with the images should simplify the use of data by those without imaging expertise (eg, by supporting studies investigating correlation of segmentation-derived features, such as volume, with nonimaging endpoints), support exploration and hypothesis generation, and enable integration of imaging phenotypes with the complementary genomics, proteomics, and other sources of data within CRDC.

### Benchmarking of the Analysis Tools

Over the past decade, biomedical image analysis challenges emerged as a mechanism to assess state-of-the-art solutions to relevant computational problems of clinical relevance. Unfortunately, most of the challenges do not require the participants to *publicly* share the easily accessible documented analysis tools or the results produced by those tools in the course of the challenge. Access to the datasets used in the challenges is often restricted to the participants in the challenge and may not be archived for persistent access. These issues limit transparency and reproducibility of the analyses performed using participating tools. Faced with a practical question of understanding and using state-of-the-art tools, the leaderboard of a challenge may be of limited value to their prospective users.

**Figure 8.**
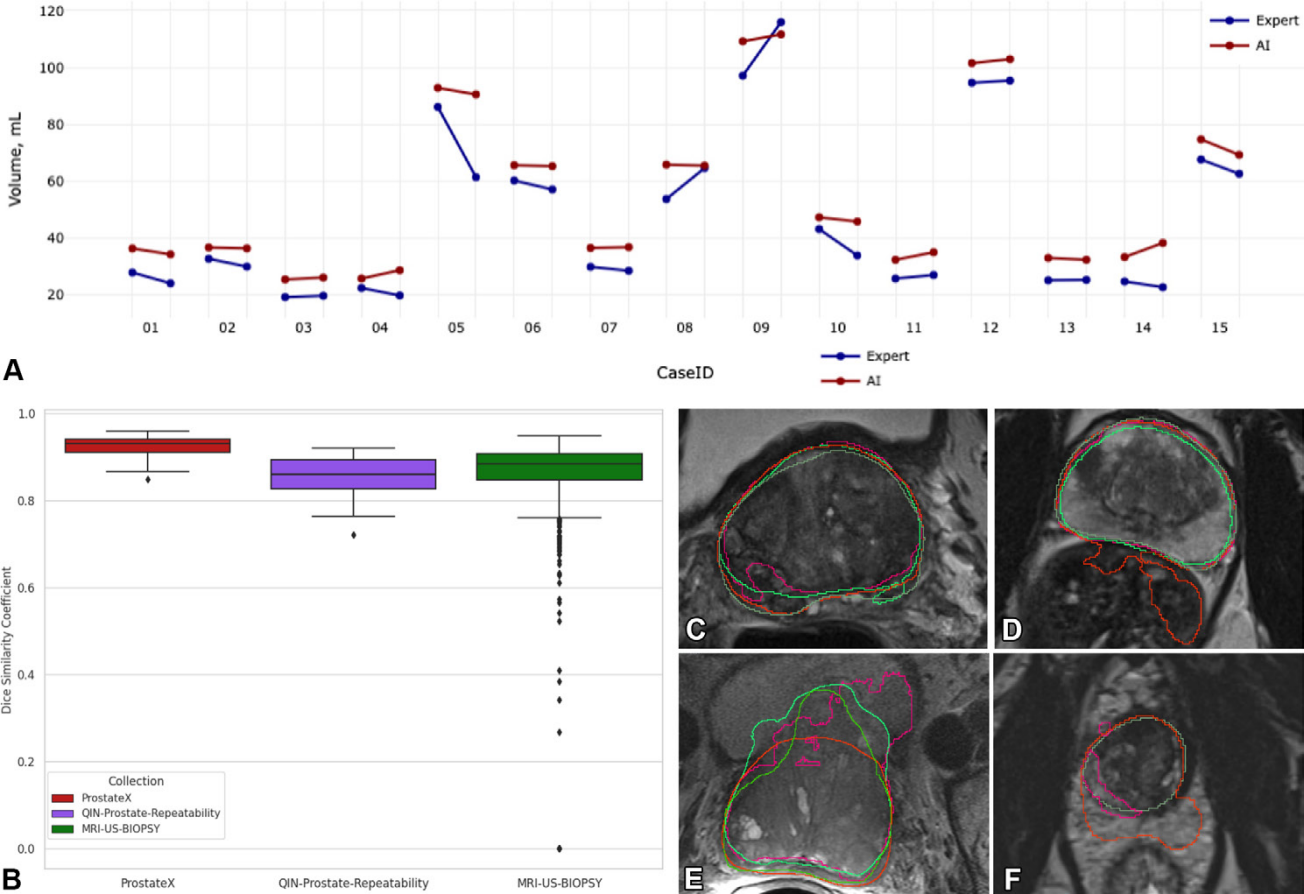
Highlights of an ongoing study evaluating the nnU-Net (42) and Prostate158 (47) algorithms applied to the prostate gland segmentation task. **(A)** Graph shows a comparison of the prostate gland volume calculated from the segmentation produced by AI (red) and the expert (blue) on the QIN-Prostate-Repeatability collection (50) available in IDC. Two measurements corresponding to the same case identification number were derived from the segmentations obtained from the two imaging studies obtained within 2 weeks, precluding biologic changes in the anatomy. Prostate volume is in most cases overestimated by AI as compared with the expert segmentations. **(B)** Dice similarity coefficient (DSC) graph shows that the distributions are visually different across the PROSTATEx, QIN-Prostate-Repeatability, and MRI-US-Biopsy collections of IDC. **(C–F)** Sample MR images from the PROSTATEx **(C, D)**, QIN-Prostate-Repeatability **(E)**, and MRI-US-Biopsy **(F)** collections show segmentation results produced by different AI algorithms and the manual outlines of the prostate (dark green).

Although, as of today, IDC does not support sequestration of data to enable algorithm challenges, it contains a growing number of annotations to help with the benchmarking efforts applied to either open-source or proprietary analysis tools. To demonstrate the potential of IDC in this area, we have been investigating open-source tools available for segmenting the prostate gland anatomy from MRI. While this topic has been the subject of extensive algorithmic development efforts for decades (44,45), a readily accessible solution to this common preprocessing task remains elusive. Utilizing expert annotations of prostate gland anatomy accompanying several IDC collections, we evaluated two open-source tools: Medical Open Network for AI (MONAI; 46) implementation of the model proposed by Adams et al (47) and nnU-Net (42) that notably showed the best performance in the Medical Imaging Decathlon challenge (48). Implementation of our ongoing benchmarking procedure is publicly available (49), and while it confirms some of the earlier findings, it also identifies limitations and problematic cases for each of the two algorithms, stimulating further refinement of the technology (Fig 8).

### Development of Enabling Technology

Computational analysis of biomedical images typically involves multiple processing steps, a variety of libraries and tools providing capabilities ranging from the visualization of images to automatic segmentation of anatomic organs, and interoperable communication of the analysis results. Development and continuous refinement of those individual components hinges on the availability of readily accessible samples of data that are representative of the complexity and variety of data encountered in practice. Permissive licenses accompanying data hosted by IDC, along with the standard interfaces to select and access representative samples based on the needs of the specific tool, support the use of IDC data for such tasks—both in the context of academic and commercial activities.

To list a few examples, data available in IDC was instrumental to improve and support the development of the open-source OHIF radiology (30) and Slim microscopy viewers (31); Bio-Formats (*https://www.openmicroscopy.org/bio-formats/*) (51), OpenSlide (*https://openslide.org/*) (52), and highdicom (*https://github.com/ImagingDataCommons/highdicom*) (53) libraries; refinements to the dcm2niix tool (*https://github.com/rordenlab/dcm2niix*), which converts from DICOM to Neuroimaging Informatics Technology Initiative (NiFTI) format (54); and commercial implementations of the DICOM standard, to name a few relevant efforts. In a recent development, Kulkarni et al (55) investigated the application of large language models (LLMs) to simplify IDC image search using the rich metadata curated by IDC.

### Large-Scale Analysis of Biomedical Data

Analysis of biomedical imaging data poses significant computational challenges, primarily due to the sheer size of both the individual images and collective cohorts and graphical processing unit hardware requirements imposed by modern AI tools. Cloud-based solutions have the ability to provide scalable computational resources on demand, with the promise of reduced costs enabled by the “pay only for what you use” model (33,56,57). In practice, adopting cloud-based solutions is not easy; there is limited evidence of proven cost benefit, while there is a potential for budget overruns in the absence of robust cost-control mechanisms.

**Figure 9.**
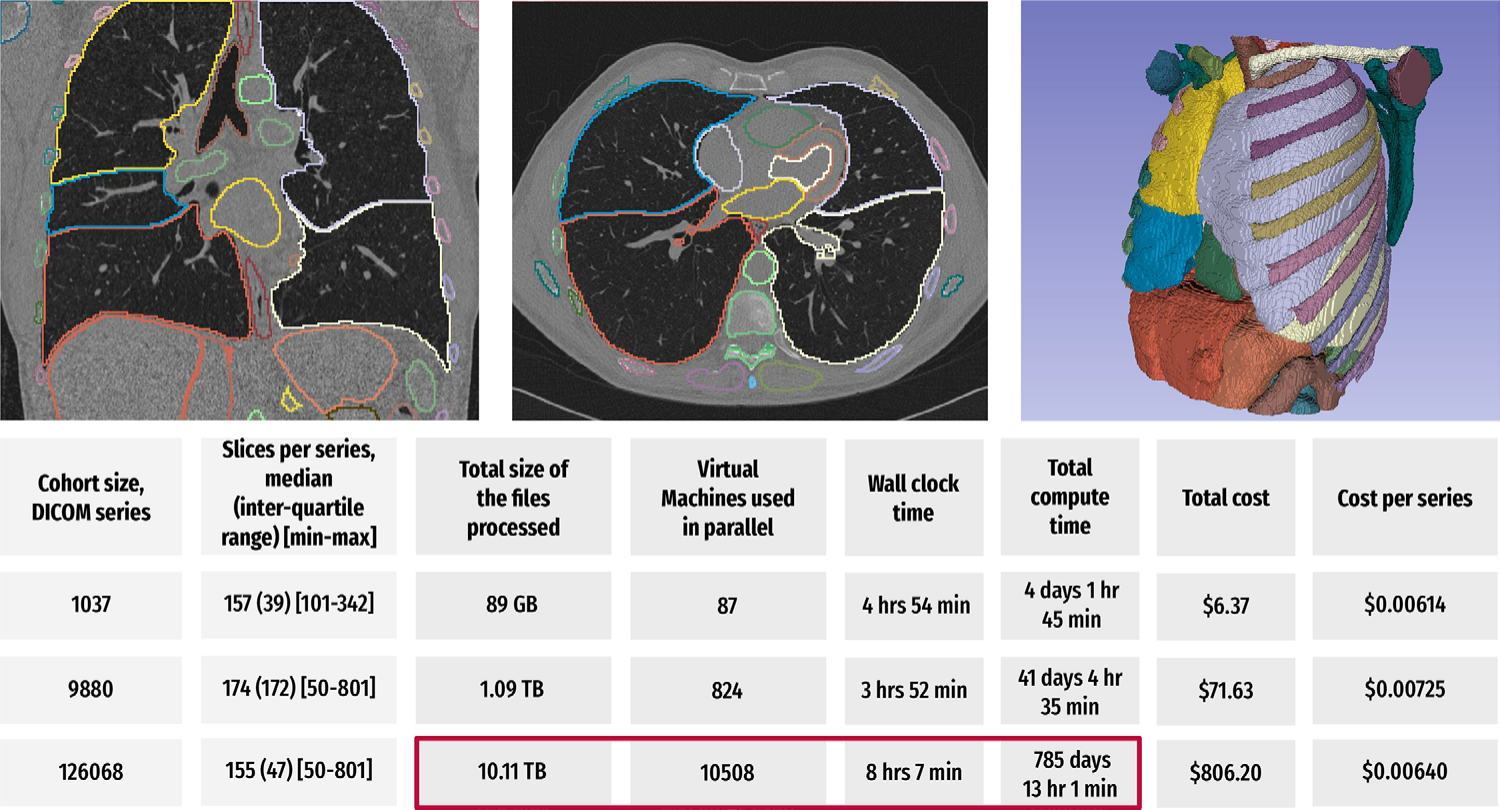
Summary of the results of a preliminary study evaluating the time- and cost-efficient scalable application of the TotalSegmentator algorithm (58) to the IDC NLST collection using CRDC resources. For each of the analyzed cases in the three cohorts of sizes 1037, 9880, and 126 068 in a CT series, the algorithm was used to segment up to 104 anatomic structures (depending on the coverage of the anatomy in a given imaging examination), followed by the extraction of the shape and first-order radiomics features for each of the segmented regions using the pyradiomics library (59). Coronal and axial CT images (top left and center, respectively) and a surface rendering of the segmentations generated using 3D Slicer (*https://slicer.org*) software (top left) show sample visualizations of the analysis (60). Bottom table summarizes the key parameters and observed performance of the two experiments. The total compute time corresponds to the time needed to perform computation sequentially. In the case of the 126 068 series analysis (red box), scaling of the processing to use 10 508 cloud-based virtual machines in parallel reduced the processing time from the estimated more than 785 days by using a single virtual machine to about 8 hours. The costs are expected to be even lower for the researchers eligible to access the discounts provided by the National Institutes of Health Science and Technology Research Infrastructure for Discovery, Experimentation, and Sustainability (STRIDES) Initiative.

Within CRDC, to help mitigate the aforementioned challenges, the Broad Institute FireCloud (*https://portal.firecloud.org/*) and Seven Bridges Cancer Genomics Cloud platforms offer additional services complementing the feature set provided by the cloud vendors, simplifying the use of the cloud. Experience using these higher-level platforms for imaging research is currently very limited. It is within the IDC mission to develop a better understanding of the best practices for using the cloud for time- and cost-efficient analysis of large cohorts. One such related initiative currently underway is focused on automatic segmentation of more than 100 anatomic structures for the entire NLST cohort using the TotalSegmentator algorithm (48) deployed via CRDC cloud resources. Our initial results demonstrate that the use of these higher-level platforms offers remarkable advantages in terms of processing time at a rather modest cost for large image cohorts, as shown in Figure 9.

## Discussion and Future Work

The NCI CRDC aims to implement a holistic approach to data curation, management, and collaborative analysis within the cancer data ecosystem. In turn, IDC should always be considered as a component within CRDC, which is intended to support all of these activities in cancer imaging research beyond serving as a data archive. Dedicated efforts of IDC to harmonize imaging and image-derived data into a uniform DICOM representation, along with the development of the supporting tools to enable such harmonization and the use of resulting data, differentiate IDC from the existing repositories, such as TCIA (9) or Medical Imaging and Data Resource Center (MIDRC) (61). Unlike project-driven repositories, where data collection procedures are formalized and defined ahead of time, such as UK BioBank (62), our goal is to accommodate a growing range of heterogeneous data collection efforts and projects, whether initially well or poorly conditioned. The data stored in IDC are available in DICOM representation as close as possible to that generated by medical imaging devices to support a broad range of uses, which is conceptually different from the approaches implemented in such repositories as Radiopaedia (63) or MedMNIST (64), where data organization and representation are targeting a very specific application.

Ingestion and harmonization of new collections of images and/or annotations and analysis results to grow the IDC data offerings remain a priority for IDC. Refinement of the procedures and development and improvement of open-source tools for conversion of user-submitted nonstandard data into DICOM representation are an active area of work. This is particularly important for submissions of whole slide digital pathology collections, where commercial adoption and deployment of DICOM in acquisition devices is in the relatively early stages (65,66).

Ease of access to the state-of-the-art AI analysis tools, and streamlining the process of applying those to the data in IDC, is another direction of ongoing development. MHub (67) is an emerging NCI-funded repository of self-contained deep learning models pretrained for a wide variety of applications and is being developed in coordination with IDC. Models curated as part of MHub are designed to be DICOM-enabled, which should ease the use of IDC and simplify contribution of the analysis results back to IDC.

There are numerous ongoing directions of work within the IDC project to address known and future challenges. One such challenge in using IDC and CRDC is the lack of experience within the community in utilizing large-scale cloud-computing resources for medical imaging data analysis tasks. Our early experience analyzing the NLST collection (at the moment, summarized only in Figure 9) demonstrates the potential for using the cloud. More work is required to document those large-scale analysis use cases and develop educational materials for the community. IDC data intake and curation currently require significant resources to review de-identification procedures implemented by the submitters, collect the accompanying metadata, and convert the data submitted into standard DICOM representation. It is expected that some of those tasks will be supported by the CRDC Data Hub, a dedicated resource within CRDC to support data submitters. Currently, IDC is limited to hosting only those collections that are available without restrictions. Subject to priorities and resources availability, we are considering adding support for limited access collections to accommodate data sequestration or limited time embargo on data release.

## Conclusion

IDC is an established “home” for findable, accessible, interoperable, and reusable (10) cancer imaging data within the national cancer data ecosystem. IDC is continuously evolving with the goal to better meet the needs of a broad community. Concerted focus on the conversion of images and image-derived data into DICOM representation empowers data exploration and enables interoperability. Balanced use of established commercial products with open-source solutions, interconnected by standard interfaces, allows us to provide value and performance, while preserving sufficient agility to address the evolving needs of the research community. Emphasis on the development of tools, use cases to demonstrate the utility of uniform data representation, and cloud-based analysis all aim to ease adoption and help define best practices. Integration with other data within the CRDC opens opportunities for multiomics studies incorporating imaging data to further empower the research community to accelerate breakthroughs in cancer detection, diagnosis, and treatment.
